# Epithelial TNF controls cell differentiation and CFTR activity to maintain intestinal mucin homeostasis

**DOI:** 10.1172/JCI163591

**Published:** 2023-10-16

**Authors:** Efren A. Reyes, David Castillo-Azofeifa, Jérémie Rispal, Tomas Wald, Rachel K. Zwick, Brisa Palikuqi, Angela Mujukian, Shervin Rabizadeh, Alexander R. Gupta, James M. Gardner, Dario Boffelli, Zev J. Gartner, Ophir D. Klein

**Affiliations:** 1 Department of Orofacial Sciences and Program in Craniofacial Biology, and; 2 Department of Pharmaceutical Chemistry and TETRAD Program, UCSF, San Francisco, California, USA.; 3 Department of Regenerative Medicine, Genentech, Inc., South San Francisco, California, USA.; 4 F. Widjaja Foundation Inflammatory Bowel and Immunobiology Research Institute, Cedars-Sinai Medical Center, Los Angeles, California, USA.; 5 Department of Pediatrics, Cedars-Sinai Guerin Children’s, Los Angeles, California, USA.; 6 Department of Surgery, and; 7 Diabetes Center, UCSF, San Francisco, California, USA.; 8 Chan-Zuckerberg Biohub, San Francisco, California, USA.; 9 The Center for Cellular Construction, San Francisco, California, USA.

**Keywords:** Gastroenterology, Cytokines, Epithelial transport of ions and water, Molecular genetics

## Abstract

The gastrointestinal tract relies on the production, maturation, and transit of mucin to protect against pathogens and to lubricate the epithelial lining. Although the molecular and cellular mechanisms that regulate mucin production and movement are beginning to be understood, the upstream epithelial signals that contribute to mucin regulation remain unclear. Here, we report that the inflammatory cytokine tumor necrosis factor (TNF), generated by the epithelium, contributes to mucin homeostasis by regulating both cell differentiation and cystic fibrosis transmembrane conductance regulator (CFTR) activity. We used genetic mouse models and noninflamed samples from patients with inflammatory bowel disease (IBD) undergoing anti-TNF therapy to assess the effect of in vivo perturbation of TNF. We found that inhibition of epithelial TNF promotes the differentiation of secretory progenitor cells into mucus-producing goblet cells. Furthermore, TNF treatment and CFTR inhibition in intestinal organoids demonstrated that TNF promotes ion transport and luminal flow via CFTR. The absence of TNF led to slower gut transit times, which we propose results from increased mucus accumulation coupled with decreased luminal fluid pumping. These findings point to a TNF/CFTR signaling axis in the adult intestine and identify epithelial cell–derived TNF as an upstream regulator of mucin homeostasis.

## Introduction

The intestine is exposed to the external environment through ingestion, and its epithelial barrier is required to prevent the entry of toxins and pathogens and restrict resident commensal microbes to the intestinal lumen (1, 2). The small intestine is folded into invaginations that house multipotent stem cells, known as crypts, and long finger-like protrusions that house differentiated cells that absorb nutrients (among other functions), known as villi. The epithelium comprises a variety of specialized cell types that derive from stem cells expressing the gene that encodes leucine-rich repeat–containing G protein–coupled receptor 5 (*Lgr5*^+^) (3). Stem cells give rise to highly proliferative absorptive and secretory progenitor cells, which in turn differentiate into cells that perform specialized functions. Absorptive progenitors produce enterocytes that absorb nutrients (1), whereas secretory progenitors give rise to Paneth, goblet, tuft, and enteroendocrine cells that release protective and regulatory factors (1). Paneth cells at the crypt bottom produce antimicrobial peptides (AMPs) that protect the epithelium from bacteria; enteroendocrine cells in the villus produce hormones that regulate digestion and absorption; tuft cells coordinate host immune responses; and goblet cells in the crypt and villus produce the majority of mucin in the intestine (4).

Goblet cell–derived mucin creates a physical protective barrier that traps pathogens and contaminants (5). In addition, the mucus layer serves as a lubricant for the passage of digested food and waste through the gut (6). Defects in mucin homeostasis, seen in diseases such as cystic fibrosis, result in accumulation of mucin, increased bacterial load, and a significant decrease in the rate of food and waste transit (7, 8). Once mucin is produced, the activity of the chloride anion channel cystic fibrosis transmembrane conductance regulator (CFTR) (9) and the protease meprin-β (10) promote mucin unfolding and shedding, respectively. While we are beginning to understand how the production and mobilization of mucin is regulated at the level of individual cells, whether and how upstream epithelial signals contribute to these processes remain unclear.

Various studies point to the cytokine tumor necrosis factor (TNF) as a potential regulator of mucin homeostasis. However, much remains to be learned, as TNF has been predominantly studied in the context of cell death and inflammation in the intestine (11, 12), and its function in nondisease states and homeostasis is less clear (13), with conflicting reports about the relationship between TNF and mucin. Contradicting studies report that TNF promotes transcription of *Muc*2 (14), the major component of mucin, while others assert that it decreases the production and thickness of the mucus layer (15, 16). Developmental studies have also shown divergent results (17, 18), and interpretation of data from in vitro studies has been limited by the use of colon cancer cell lines, which lack intestinal cell type diversity and crypt-villus architecture (19). Thus, the role of TNF in adult intestinal homeostasis and mucin production, and the cell types that produce and respond to TNF signals, are unknown.

In this study, we aimed to elucidate the cellular source and functional role of TNF during mucin homeostasis by examining mouse and human adult intestinal tissues and using intestinal organoids that more closely recapitulate in vivo physiology than do 2D cell line models (20). RNA in situ hybridization and immunohistochemical analysis revealed that crypt cells are the major epithelial producers of TNF. Genetic studies demonstrated that loss of TNF led to mucin accumulation and slower gut transit time. By combining genetic ablation of *Tnf* with lineage tracing of progenitor cells, we found that TNF is required for maintaining the proportion of secretory and absorptive progenitors and for suppressing a goblet cell differentiation bias. Using live imaging, we determined that TNF regulates luminal fluid pumping in organoids by modulation of CFTR activity, establishing a TNF/CFTR signaling axis for mucin flux in the intestine. Finally, to establish the clinical relevance of our mouse and organoid studies to human physiology, we obtained ileal samples from healthy human donors, patients with inflammatory bowel disease (IBD), and IBD patients treated with anti-TNF therapy and performed immunohistochemical analysis of goblet cells. Patients treated with anti-TNF therapy had a higher proportion of goblet cells when compared with noninflamed IBD samples or even healthy donor samples. This closely matched the increase in goblet cell number we observed when epithelial TNF was deleted in mouse intestine and suggests that anti-TNF therapy–induced goblet cell hyperplasia may similarly lead to increased mucin accumulation, bacterial load, and small intestinal transit. We therefore conclude that TNF contributes to mucin homeostasis by regulating both secretory cell differentiation into goblet cells and mucin maturation through the activity of CFTR. Our data identify a previously underappreciated TNF/CFTR signaling axis in intestinal homeostasis and disease.

## Results

### TNF, TNFR1, and TNFR2 are expressed in defined spatial domains along the crypt-villus axis.

**Figure 1:**
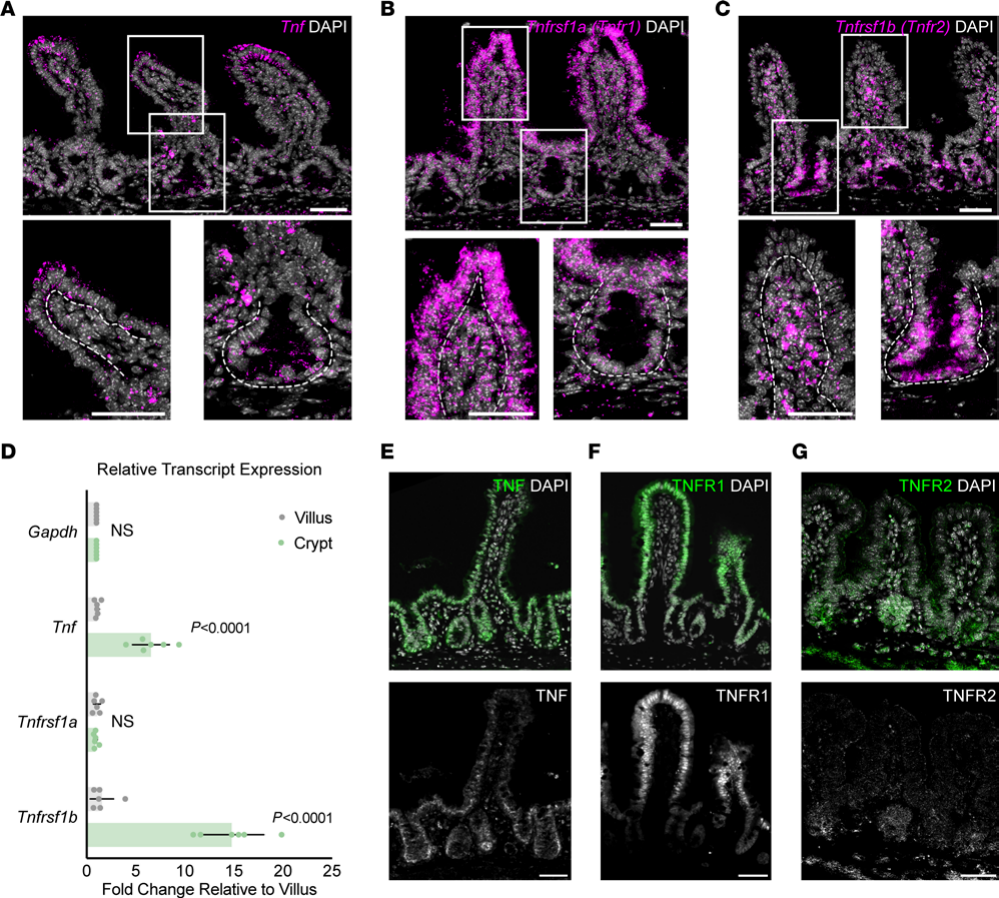
TNF and its receptors are expressed in defined spatial domains within epithelial crypts and villi. (**A**–**C**) RNAscope of *Tnf*, *Tnfrsf1a* (*Tnfr1*), and *Tnfrsf1b* (*Tnfr2*). (**A**) *Tnf* transcripts localize to the villus tip and the crypt base. (**B**) *Tnfrsf1a* transcripts are broadly expressed in the epithelium. (**C**) *Tnfrsf1b* transcripts are enriched in epithelial crypts compared with villi. White boxes highlight insets. Dashed lines outline the epithelium-mesenchyme border. (**D**) qPCR analysis of cells sorted by CD44 expression, CD44^hi^ = crypt cells, CD44^lo^ = villus cells. *P* values were calculated by unpaired multiple *t* test analysis with Welch’s correction (*n* = 6 mice per group). (**E**–**G**) TNF, TNFR1, and TNFR2 immunofluorescence in control mice. (**E**) TNF is broadly present in the epithelium, with an enrichment in crypts. (**F**) TNFR1 is highly present in the top of the villus epithelium and gradually decreases toward the bottom of the crypt. (**G**) TNFR2 is enriched in crypts. Scale bars: 50 μm.

To identify possible sender-receiver signaling relationships between cells in the small intestine, we assessed the mRNA and protein expression of the ligand TNF and its receptors TNFR1 and TNFR2. We utilized probes for single-molecule RNA in situ hybridization (RNAscope) targeting *Tnf* (encodes TNF), *Tnfrsf1a* (encodes TNFR1), and *Tnfrsf1b* (encodes TNFR2) to visualize mRNA expression in the adult intestine (Figure 1, A–C). *Tnf* expression was enriched at the villus tip and epithelial crypt (Figure 1A), *Tnfrsf1a* was broadly expressed with an enrichment in villi (Figure 1B), and *Tnfrsf1b* was strongly enriched in the crypt epithelium (Figure 1C). By FACS isolating epithelial crypt and villus cells from the mouse ileum and performing qPCR, we quantitatively compared the expression of *Tnf*, *Tnfrsf1a*, and *Tnfrsf1b* in both intestinal compartments (Figure 1D). Compared with villus cells, crypt cells expressed approximately 6-fold more *Tnf,* comparable *Tnfrsf1a,* and 15-fold more *Tnfrsf1b* transcripts, closely matching the RNAscope data.

We next examined protein abundance using immunohistochemistry and found similar protein localization along the crypt-villus axis (Figure 1, E–G). TNF was broadly present in intestinal epithelium and enriched in epithelial crypts (Figure 1E). TNFR1 expression was highest at the villus tip and gradually decreased toward the crypt (Figure 1F) and TNFR2 was strongly enriched in crypts (Figure 1G). When we deleted *Tnf* (Supplemental Figure 1A; supplemental material available online with this article; https://doi.org/10.1172/JCI163591DS1), *Tnfrsf1a* (Supplemental Figure 1B), or *Tnfrsf1b* (Supplemental Figure 1C), expression of the proteins encoded by these genes was no longer detected, validating the in situ and immunofluorescence results and demonstrating that the genetic tools are robust.

These findings demonstrate a defined expression pattern of TNF ligand and its receptors, which may permit different cellular responses to the same TNF signal across the crypt-villus axis.

### The absence of TNF causes increased luminal mucin, goblet cell number, gut transit time, and bacterial load.

**Figure 2:**
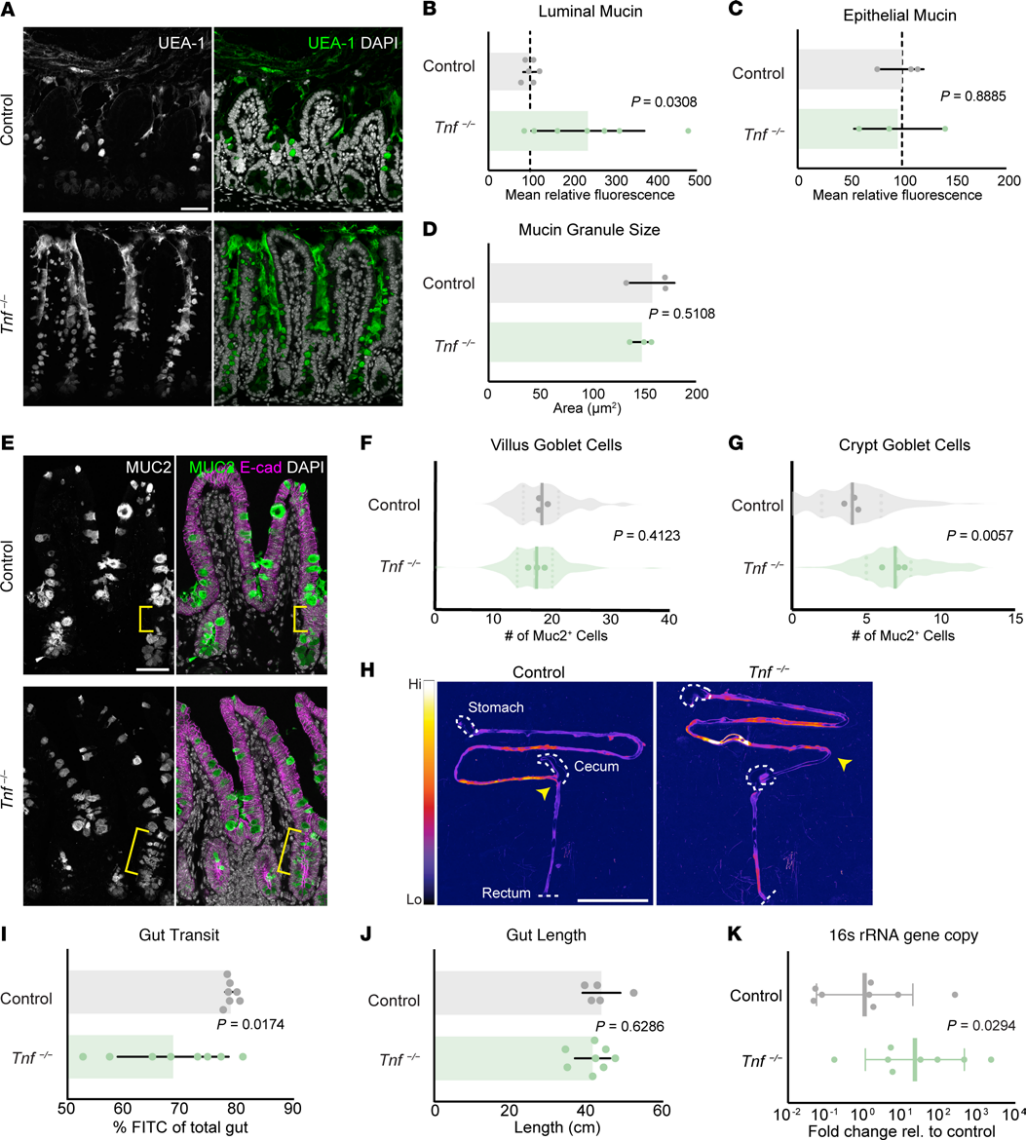
TNF-null intestines have increased luminal mucin, goblet cell numbers, intestinal transit time, and bacterial load. (**A**) Mucin fluorescence by UEA-1 lectin staining in adult intestine. Scale bar: 200 μm. (**B**) Quantification of mean fluorescence intensity of intervillus regions (*n* = 6 mice per group, 15 regions of interest per mouse). (**C**) Quantification of MUC2 fluorescence intensity per goblet cell (*n* = 3 mice per group). Mean represents all goblet cells in the distal 10 cm segment of a sectioned intestine. Values are normalized to mean of control. (**D**) Mucin granule size calculated as the mean fluorescence area of MUC2 signal in cells of the same regions of interest marked in panel **C**. (**E**) Immunostaining of MUC2^+^ goblet cells. Yellow brackets indicate MUC2^+^ goblet cells above Paneth cell zone in crypts. Scale bar: 50 μm. (**F** and **G**) Quantification of goblet cells in crypts and villi of mice (*n* = 3 mice per group, 30 full profile crypt-villus units counted per mouse). (**H**) Micrographs of full-length intestines showing FITC displacement after 1 hour. Arrows indicate the most distal FITC signal. Dashed lines outline tissue landmarks. Scale bar: 5 mm. (**I**) Quantification of FITC displacement represented as percentage of gut length, i.e., FITC signal over total gut length (*n* = 6 control, 7 *Tnf^–/–^* mice). (**J**) Total gut length was quantified as the distance from the stomach to the rectum (*n* = 5 control, 8 *Tnf^–/–^* mice). (**K**) Relative bacterial load in feces collected from the ileum of mice represented by the fold change in 16S rRNA gene copies compared with control (*n* = 8 mice per group). *P* values calculated by unpaired, 2-tailed *t* test (**B**–**D**, **F**, **G**, **I**, and **J**) or by unpaired, 1-tailed *t* test (**K**).

Elevated levels of TNF in Crohn’s disease tissue samples or treatment of colon cell lines with exogenous TNF are associated with decreased abundance and thickness of the mucus barrier (16, 21, 22), suggestive of a role for TNF in maintaining intestinal mucus. However, whether and how TNF might regulate mucin production under homeostatic conditions is unclear. Therefore, we evaluated the role of TNF in mucin homeostasis by analyzing constitutive *Tnf^–/–^* mice (23), which lack TNF in all tissues, including the intestinal epithelium. In comparison with age-matched control mice, luminal mucin was elevated in *Tnf^−/−^* mice (Figure 2, A and B). However, intracellular epithelial mucin measured by mean mucin fluorescence (Figure 2C) and granule size per goblet cell (Figure 2D) were unaffected. These findings suggested that increased luminal mucin levels in mutant mice could be explained, at least in part, by an increase in mucus-producing goblet cells. We then quantified goblet cell number in control and mutant villi and crypts and found that, while the number of goblet cells remained constant in villi (Figure 2, E and F), mutant mice had more goblet cells in crypts (Figure 2, E and G).

Prior work has correlated increased luminal mucin with increased gut transit time (8). Therefore, we tested whether gut transit time was delayed in *Tnf^−/−^* mice. Using a 70 kDa FITC-dextran dye, we tracked anteroposterior dye displacement along the gut length over time (Figure 2H). After 1 hour, FITC-dextran traveled 80% of the total gut length of control mice and 70% of the total gut of mutant mice (Figure 2I), while total gut length remained constant between both groups (Figure 2J). These findings indicate that, in the absence of TNF, the expulsion of digested food and waste is impaired.

Together with slower gut transit, previous work has shown that increased luminal mucin is associated with higher levels of bacterial load (24). We assessed the bacterial load of control and mutant mice by isolating genomic DNA (gDNA) from feces and performing qPCR for the universal bacterial 16S rRNA gene, which showed that bacterial load in mutant mice was elevated approximately 20-fold compared with control (Figure 2K). Thus, during homeostasis, TNF contributes to the regulation of intestinal mucin levels, goblet cell number, gut transit, and bacterial load.

### TNF does not affect secretory cell turnover, but controls goblet cell number by regulating secretory progenitor cell differentiation.

Goblet cell number depends on the rate of loss of mature goblet cells through cell death and/or extrusion from the villi and the rate of differentiation from secretory progenitors. We investigated how goblet cell numbers increase in the crypts of *Tnf^−/−^* mice using a lineage tracing approach, focusing on the fate of secretory progenitors. We bred *Atoh1^CreERT2^; Rosa26^tdTomato^* mice (25–27), which label secretory progenitors upon tamoxifen induction, with *Tnf^−/−^* mice, and then lineage traced secretory progenitors in the absence of TNF.

**Figure 3:**
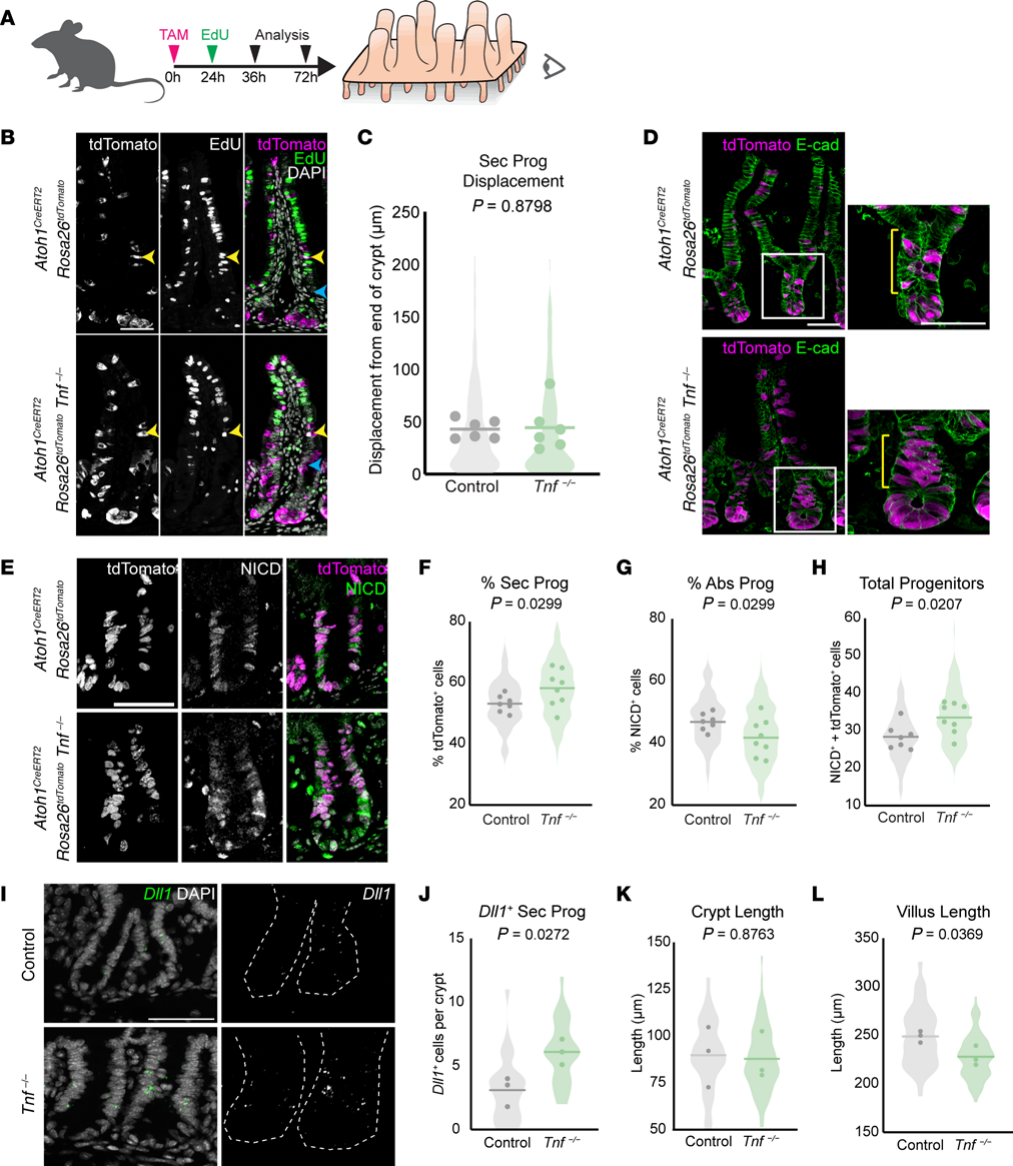
TNF does not affect secretory cell turnover, but regulates the proportion of absorptive and secretory progenitors. (**A**) Experimental design. TAM, tamoxifen. (**B**) Micrograph of *Atoh^CreERT2^; Rosa26^tdTomato^* control and *Tnf^–/–^* mice induced with tamoxifen at 0 hours to label secretory progenitors, injected with EdU at 24 hours to label proliferating cells, and analyzed at 72 hours. Yellow arrows mark tdTomato^+^EdU^+^ cells. Cyan arrows pinpoint the hinge region. (**C**) Quantification of secretory progenitor daughter cell (tdTomato^+^EdU^+^) displacement from the hinge region (*n* = 6 mice per group, 25 full-profile crypt-villus units per mouse). (**D**) Micrographs of *Atoh^CreERT2^; Rosa26^tdTomato^* control and *Tnf^–/–^* mice induced with tamoxifen for 36 hours. White boxes highlight insets. Yellow brackets show the progenitor zone. (**E**) Representative crypts labeled for both absorptive (NICD^+^) and secretory (tdTomato^+^) progenitors. (**F**) Percentage secretory progenitors and (**G**) percentage absorptive progenitors were quantified as the number of tdTomato^+^ or NICD^+^ cells, respectively, over total progenitors (NICD^+^ and tdTomato^+^, shown in **H**) from the +4 region to the top of the crypt (*n* = 7 control, 8 *Tnf^–/–^* mice, with 20 full-profile crypts per mouse). (**I**) Representative RNAscope micrograph of *Dll1* transcripts in control and *Tnf^–/–^* mice. (**J**) *Dll1^+^* secretory progenitors were quantified by counting high-*Dll1*-expressing cells (with more than 3 dots per cell) from the +4 region to the top of the crypt (10 crypts per mouse, *n* = 3 mice per group). (**K** and **L**) Crypt length was measured as the distance from the bottom of the crypt to the hinge region. Villus length was measured as the distance from hinge region to villus tip (*n* = 3 mice per group, at least 30 full-profile crypts were measured per mouse). *P* values calculated by unpaired, 2-tailed *t* test (**C**, **F**–**H**, and **J**–**L**). Scale bars: 50 μm.

Crypt cells give rise to differentiated cells that transit along the crypt-villus axis to replace more mature cells that ultimately die and are shed at villus tips (27). This continuous epithelial turnover takes approximately 3–5 days in the small intestine (1, 28). We predicted that lower rates of differentiated cell turnover would lead to an increase in goblet cell number due to the persistence of differentiated goblet cells in the epithelium. To explore this possibility, we measured the movement of labeled proliferating cells out of the crypt in *Atoh1^CreERT2^*; *Rosa26^tdTomato^*; *Tnf^−/−^* mice as a proxy for secretory cell turnover (27, 29). We treated mutant mice with a single tamoxifen dose followed by a single injection of the thymidine analog EdU 24 hours later (Figure 3A). Over the course of 72 hours, proliferative secretory progenitors were uniquely labeled with both tdTomato and EdU, enabling us to map the appearance of nascent mature secretory cells arising from secretory progenitors and to measure the movement of double-positive tdTomato^+^EdU^+^ cells out of the crypt and up the villus. We found that after 48 hours of EdU labeling, control and mutant secretory progenitor cells alike were displaced approximately 50 μm from the hinge region that separates the crypt and villus compartments (Figure 3, B and C), suggesting that secretory cell turnover was unaltered.

Next, we assessed the number of secretory progenitors in crypts, which would affect the overall rate of differentiation into goblet cells. In our previous experiments, both control and mutant secretory progenitors were similarly displaced approximately 50 μm from the hinge region over the course of 48 hours. Therefore, we chose to chase for 36 hours to preferentially label secretory progenitors while minimizing lineage tracing of differentiated secretory cells in the villus. We induced *Atoh1^CreERT2^*; *Rosa26^tdTomato^*; *Tnf^−/−^* mice with a single tamoxifen pulse followed by a short chase of 36 hours (Figure 3A). We found that labeled *Atoh1^CreERT2^*; Rosa26*^tdTomato^*; *Tnf^−/−^* mice had increased numbers of tdTomato^+^ cells within crypts. The increase in the number of tdTomato^+^ cells indicates an increase in ATOH1^+^ secretory progenitors, supporting a role for a secretory differentiation bias in the absence of TNF (Figure 3D). Since the crypt houses secretory (ATOH1^+^) and absorptive (NICD^+^) progenitors (26), we next assessed whether the increase in ATOH1^+^/tdTomato^+^ secretory progenitors was specific to this lineage and at the expense of the number of absorptive progenitors by staining absorptive progenitors for NICD (Figure 3E). We found that *Tnf^−/−^* mice have an increase in tdTomato^+^ secretory progenitors, with a corresponding decrease in absorptive progenitors (Figure 3, F and G). To determine whether there was a specific increase in secretory progenitors and not a general increase in all cells, we used crypt and villus length to estimate any changes in total cell number. We found that the total number of progenitors per crypt increased (Figure 3H), crypt length was unchanged (Figure 3K), and villus length was shorter (Figure 3L), consistent with a biased increase in secretory progenitors. To verify that the increase in ATOH1^+^ cells represents an increase in secretory progenitors and not goblet cells labeled during lineage tracing, we stained for the bona fide secretory progenitor marker *Dll1* (30) and found that *Tnf^−/−^* mice showed a significant increase in the number of cells with *Dll1* transcripts, again consistent with an increase in secretory progenitors (Figure 3, I and J).

### TNF expressed by epithelial cells regulates mucin homeostasis and goblet cell differentiation.

**Figure 4:**
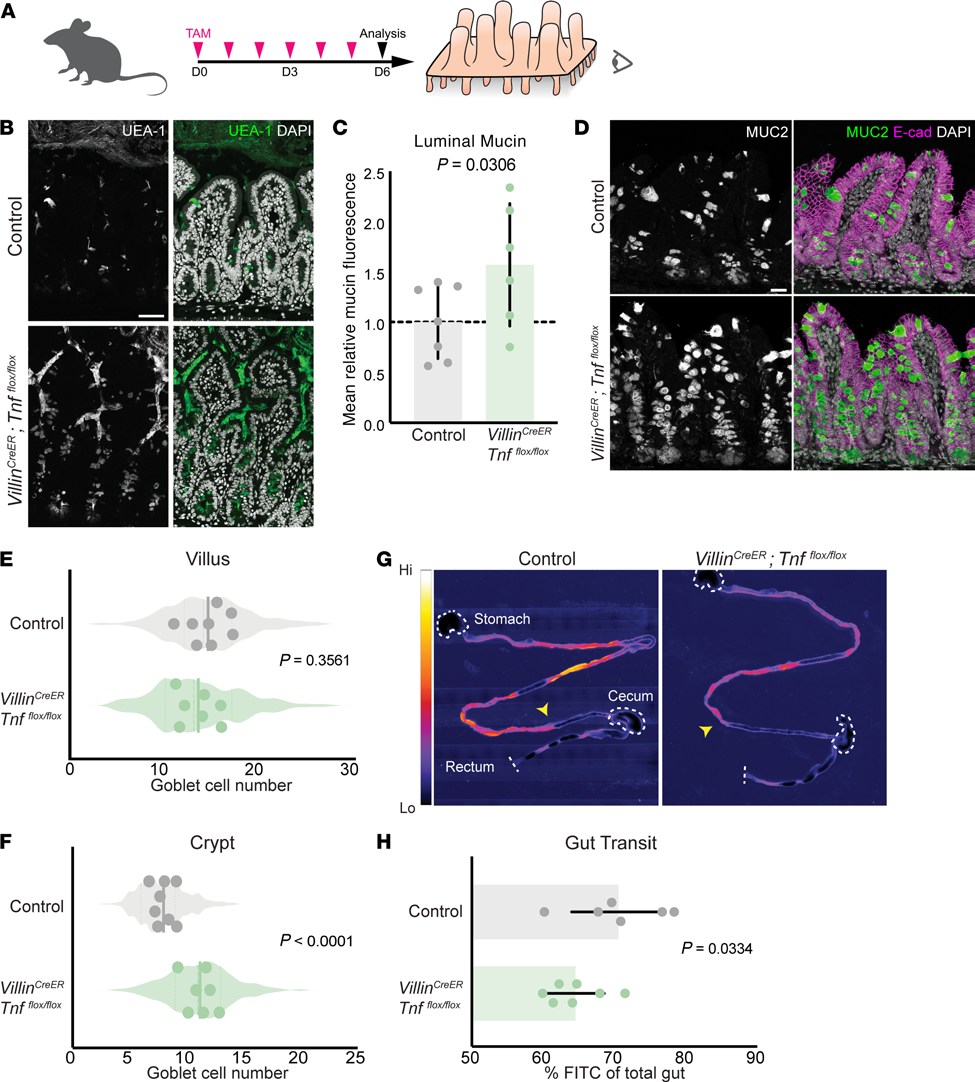
Epithelium-derived TNF is required for mucin and goblet cell homeostasis. (**A**) Experimental design. TAM, tamoxifen. (**B**) Mucin fluorescence by UEA-1 lectin staining in adult tissue. Note that *Villin^CreERT2^; Tnf^fl/fl^* mice have stronger staining intensity and more luminal area covered by mucin. Scale bar: 50 μm. (**C**) Quantification of mean fluorescence intensity of intervillus regions. Values are normalized to mean fluorescence of controls (*n* = 6 control, 7 *Villin^CreERT2^; Tnf^fl/fl^* mice, 20 regions of interest per mouse). (**D**) Micrographs of MUC2^+^ goblet cells in the intestine. Scale bar: 50 μm. (**E** and **F**) Quantification of MUC2^+^ goblet cells (*n* = 5 control, 6 *Villin^CreERT2^; Tnf^fl/fl^* mice, 25 crypt-villus units per mouse). (**G**) Micrographs of FITC displacement after 1 hour across full-length intestines. Arrows indicate most distal FITC signal. Dotted lines outline tissue landmarks. Scale bar: 2 mm. (**H**) Quantification of FITC displacement represented as percentage of gut length, i.e., FITC signal over total gut length (*n* = 6 control, 7 *Villin^CreERT2^; Tnf^fl/fl^* mice). *P* values calculated by unpaired, 2-tailed *t* test (**C**, **E**–**F**, and **H**).

Prior work (31, 32) and our own immunohistochemical analysis of TNF suggests that the epithelium is a significant source of TNF that drives the observed mucin phenotypes. However, it has been shown that mesenchymal cells are also an intestinal source of TNF (33). To determine whether epithelial cell–derived TNF is required for proper mucin homeostasis, we generated mice harboring both the *Vil^CreERT2^* and *Tnf^fl/fl^* alleles (34, 35), which allowed for spatiotemporal deletion of TNF, as opposed to constitutive deletion in *Tnf^−/−^* mice. In *Vil^CreERT2^*; *Tnf^fl/fl^* mice, we deleted TNF in intestinal epithelial cells by administering daily doses of tamoxifen over 6 days, which is approximately 2 epithelial turnover cycles (Figure 4A). Acute epithelial loss of TNF phenocopies the effects observed during the constitutive loss of TNF. While *Vil^CreERT2^*; *Tnf^fl/fl^* mutants showed no difference in gut length compared to controls (Supplemental Figure 1, D–F), mutant mice had increased luminal mucin (Figure 4, B and C). We also observed higher goblet cell numbers (Figure 4, D and F) in the crypt (Figure 4F), while villus (Figure 4E) and total goblet cell numbers remained unchanged (Supplemental Figure 1D). To determine whether acute epithelial loss of TNF also caused defects in waste expulsion, we performed a gut transit assay. After 6 days of tamoxifen induction, we measured the distance that FITC-dextran traveled along the gastrointestinal tract over 1 hour. The dye traveled 6% less of the total gut in mutant mice compared with controls (Figure 4, G and H), similar to what we observed in *Tnf^−/−^* mice. Taken together, these studies establish a central role for epithelial cell–derived TNF in regulating mucin homeostasis by inhibiting secretory progenitor bias to goblet cells.

### Epithelial TNF-TNFR1 interactions control mucin flux via CFTR-induced fluid pumping.

Several phenotypes observed in our *Tnf^−/−^* mice resembled those previously reported in *Cftr^−/−^* mice, including increased intestinal mucin levels, goblet cell number, gut transit, and bacterial load (7, 8, 24). CFTR function is essential for proper mucin flux and maturation in the intestine (9, 10). We therefore asked whether epithelial TNF acted as an upstream regulator of CFTR, in addition to regulating epithelial cell differentiation. We first characterized the spatial expression of CFTR within the intestine using RNAscope and found that *Cftr* transcripts were spatially restricted to epithelial crypts in the ileum and a subpopulation of villus cells. Among cells in the crypt, LGR5^+^ stem cells highly expressed *Cftr* (Supplemental Figure 2A). These data are consistent with previous reports of CFTR expression (36–38) and suggest that the crypt base is a major site of fluid and ion pumping downstream of CFTR activity. Furthermore, the zonated expression of CFTR, TNF, and TNFRs (Figure 1) in epithelial crypts indicate a potential interaction between CFTR and TNF in crypts.

**Figure 5:**
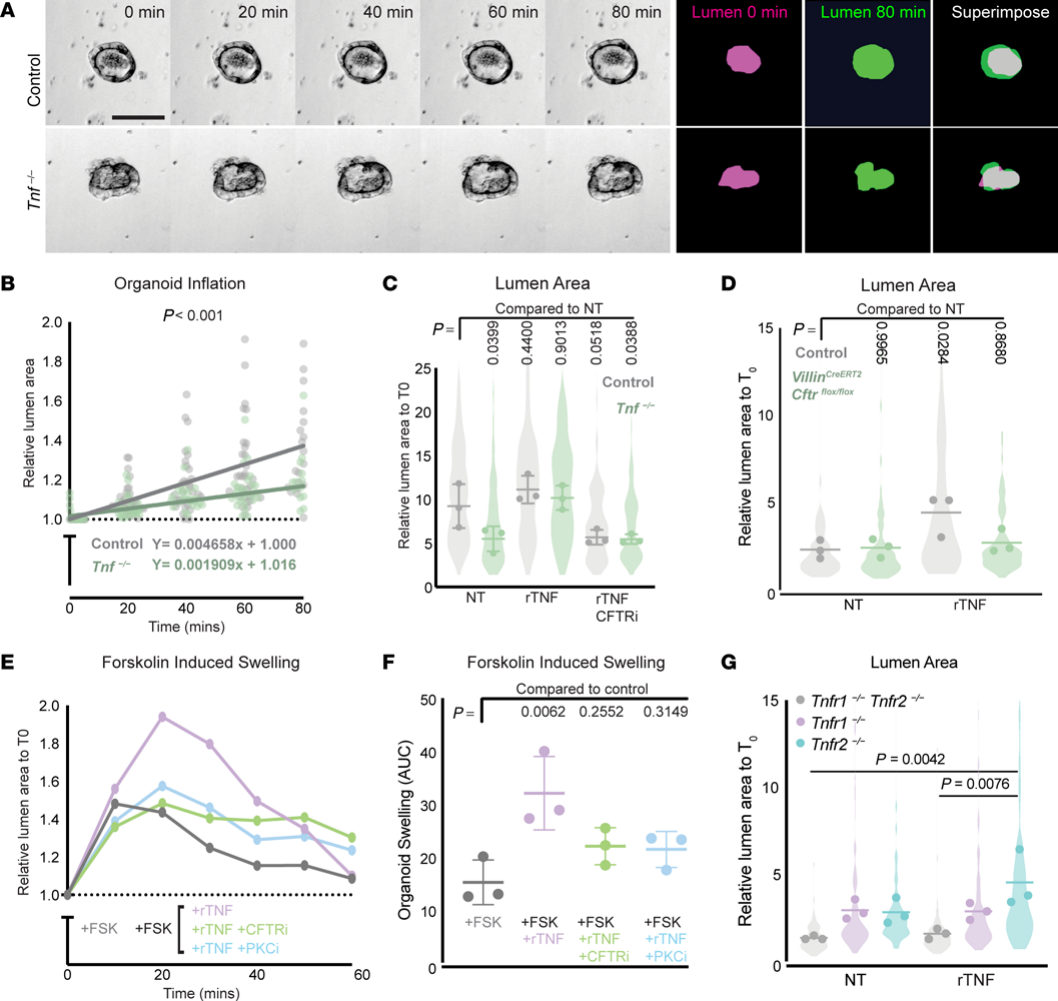
Epithelial TNF modulates CFTR-induced fluid pumping through TNFR1. (**A**) Eighty-minute montage of WT and *Tnf^–/–^* organoids live imaged 8 hours after plating under normal growth conditions. Magenta masks outline lumens at 0 minutes and green masks outline lumens at 80 minutes. Scale bar: 50 μm. (**B**) Quantification of the inflation rate of organoids during 80 minutes without lumen collapse under normal growth conditions. Lumen size was normalized to *t* = 0 minutes and was measured for each individual organoid at 20-minute intervals. A simple linear regression was performed for compiled data. (**C**) Lumen size of organoids treated with 1 ng/mL rTNF and 50 μM CFTRinh-172 for 24 hours. Measurements were normalized to the size of individual lumens at *t* = 0 hours. NT, no treatment. (**D**) Lumen size of control or *Villin^CreERT2^; Cftr^fl/fl^* organoids treated with 1 ng/mL rTNF for 24 hours. (**E**) Lumen swelling of 2-day-old WT organoids costimulated with 0.4 μM forskolin and 1 ng/mL rTNF plus inhibitors of either CFTR (20 μM CFTRinh-172) or PKC (5 μM GF109203X). Plots represent mean of 30 organoids from 3 distinct organoid lines. FSK, forskolin. (**F**) Area under the curve of forskolin-induced swelling plots. Each point represents the mean AUC for an individual organoid line within the group. (**G**) Lumen size of control or *Tnfr*-KO organoids treated with 1 ng/mL rTNF for 24 hours. For all organoid experiments, *n* = 3 distinct established organoid lines per group with more than 20 organoids quantified per line. A 2-tailed *P* value was calculated testing the null hypothesis that the slopes between the 2 groups were equal in **B**. *P* values were calculated using an ordinary 1-way ANOVA with Dunnett’s multiple-comparison test comparing all groups to the control for **C**, **D**, and **F** and an ordinary 1-way ANOVA with Turkey’s multiple-comparison test for **G**.

Intestinal organoids provide an ideal system to dissect molecular mechanisms intrinsic to the epithelium by enabling the analysis of genetic and pharmacological perturbations coupled with live imaging. During normal organoid growth, fluid flows into organoid lumens and causes them to periodically inflate and collapse in a CFTR-dependent manner (39). Indeed, inflation of organoids has been used to predict patient response to drugs in cystic fibrosis, which is caused by loss-of-function mutations in CFTR (40, 41). We assessed the impact of TNF on CFTR activity by first measuring the rates of inflation of control and *Tnf^−/−^* organoids cultured in normal growth conditions. The rate of inflation of *Tnf^−/−^* organoids was significantly reduced in normal growth media, approximately 2.5 times slower than controls (Figure 5, A and B). Consistent with a role for TNF upstream of CFTR, recombinant TNF (rTNF) rescued the lumen size of *Tnf^−/−^* organoids at 24 hours of growth, and combined treatment with rTNF and a CFTR inhibitor (CFTRinh-172) blocked the previously observed rescue (Figure 5C). Using a *Villin^CreERT2^; Cftr^fl/fl^* conditional knockout (8), we were able to further validate that TNF acts upstream of CFTR to induce luminal flow, as *Cftr* loss of function reversed the organoid inflation induced by rTNF treatment (Figure 5D).

We next aimed to determine the mechanism by which TNF modulates CFTR activity. We hypothesized that TNF could increase the total amount of CFTR and thus increase CFTR activity. To test this, we performed qPCR on control or *Tnf^−/−^* organoids grown for 24 hours in culture. We found that there was no change in *Cftr* transcripts in *Tnf^−/−^* organoids (Supplemental Figure 2C), suggesting that TNF acts on CFTR posttranscriptionally. Therefore, we hypothesized that rTNF was increasing the quantity of active CFTR in organoids. To obtain a more accurate readout of maximal CFTR function, we used forskolin to elevate cyclic AMP levels and stimulate CFTR activity and swelling in intestinal organoids (40). Previous work showed that rTNF stimulates CFTR activity through a PKC-dependent mechanism in human bronchial epithelial cells (42). Therefore, we costimulated control organoids with forskolin and combinations of rTNF, CFTRinh-172, and the PKC inhibitor bisindolylmaleimide I (GF109203X). We found that TNF had an additive effect with forskolin in inducing organoid swelling and caused an overall 2-fold increase in lumen swelling. This additive effect was abrogated with CFTR inhibition, confirming that rTNF-induced swelling acts through CFTR. Additionally, rTNF cotreatment with the PKC inhibitor GF109203X abrogated the additive effect of rTNF, pointing to a PKC-dependent mechanism (Figure 5, E and F). Thus, we conclude that rTNF modulates CFTR-induced fluid pumping through PKC.

To identify whether TNFR1 or TNFR2 was required for TNF modulation of CFTR-induced flow, we generated single- (*Tnfr1^−/−^*, *Tnfr2^−/−^*) and double-knockout (*Tnfr1^−/−^ Tnfr2^−/−^*) organoids and assessed their ability to swell following rTNF treatment. As expected, *Tnfr1^−/−^ Tnfr2^−/−^* double-knockout organoids did not show a significant change in lumen size after 24 hours of rTNF treatment (Figure 5G). We then compared swelling in *Tnfr1^−/−^* and *Tnfr2^−/−^* organoids to swelling in *Tnfr1^−/−^ Tnfr2^−/−^* double-knockout organoids. At baseline, single *Tnfr1^−/−^* and single *Tnfr2^−/−^* organoids showed a trend toward increased lumen size compared with *Tnfr1^−/−^ Tnfr2^−/−^* double-knockout organoids without treatment, which suggests that TNFR1 and TNFR2 each partially contribute to lumen size during homeostasis. Nevertheless, *Tnfr1^−/−^* organoids did not show a statistically significant change in lumen size after 24 hours of rTNF treatment, whereas *Tnfr2^−/−^* organoids showed a statistically significant increase in lumen size after 24 hours of rTNF treatment (Figure 5G), suggesting that TNF acts primarily through TNFR1 to modulate CFTR-induced flow.

Ions released by CFTR promote mucin unfolding, exposing sites where the protease meprin-β can then cleave and thereby allow mucin to be processed and released (5). Failure to shed meprin-β from epithelial cell membranes leads to unprocessed MUC2 and more dense mucus (10). Therefore, we asked whether epithelial TNF also acts upstream of meprin-β and assessed the activity of meprin-β in *Vil^CreERT2^*; *Tnf^fl/fl^* mice by using protease localization as a readout. *Vil^CreERT2^*; *Tnf^fl/fl^* mice showed more intense and dense meprin-β staining compared with controls (Supplemental Figure 2), indicative of less shed meprin-β and consequently a decreased ability to cleave mucin. Combined, these results expand the role of epithelial TNF in mucin homeostasis beyond control of goblet cell number to include modulation of 2 key processes in mucin flux: regulation of CFTR activity and meprin-β shedding.

### IBD patients treated with anti-TNF have an increased number of goblet cells in crypts.

**Figure 6:**
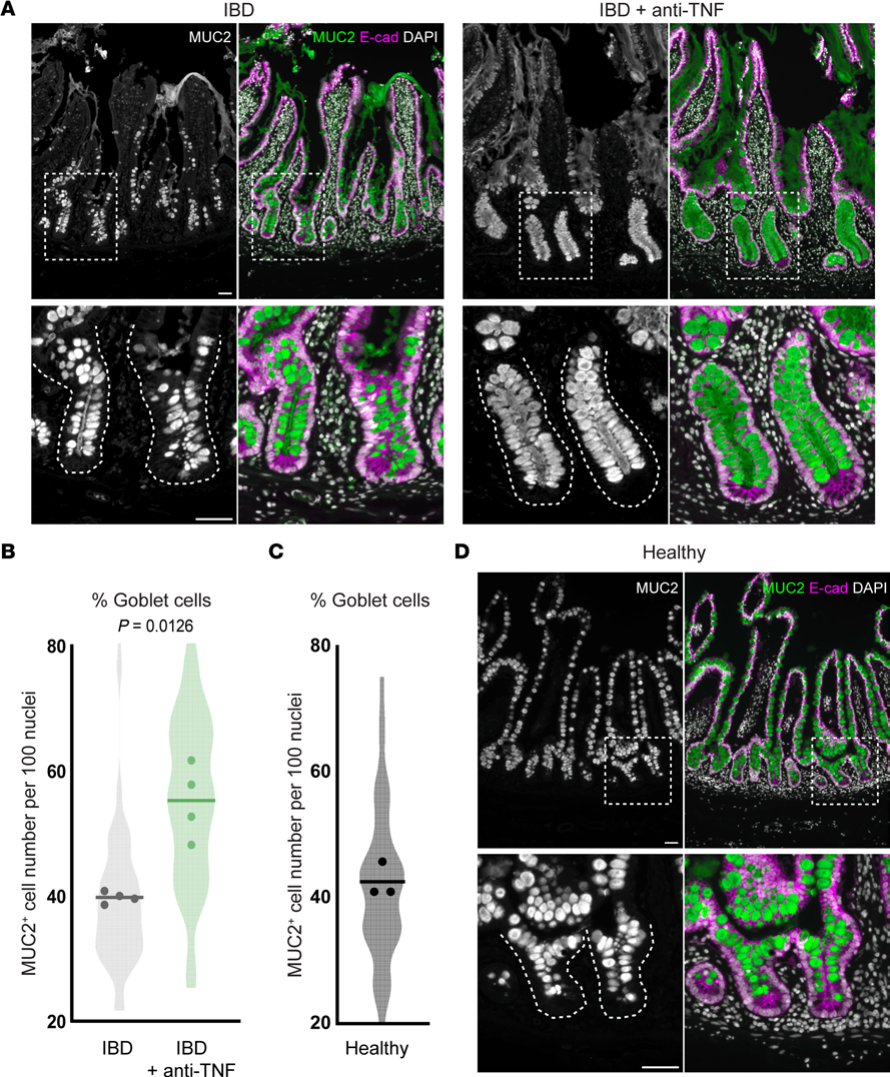
Anti-TNF treatment in human IBD patients triggers goblet cell hyperplasia in crypts. (**A**) Representative immunostaining of MUC2^+^ goblet cells in human ileal sections from patients diagnosed with IBD and either untreated or treated with anti-TNF therapy. The epithelium is stained for E-cadherin. (**B**) The proportion of goblet cells per crypt was quantified by dividing the number of MUC2^+^ cells per the total number of nuclei in each crypt. *P* value was calculated by unpaired, 2-tailed *t* test with Welch’s correction (*n* = 4 individuals per group, 10–20 different crypts were counted for each individual). (**C** and **D**) Quantification of the proportion of goblet cells per crypt and representative images of goblet cells in the Ileum of healthy donors (*n* = 3 individuals, 10–20 different crypts were counted for each individual). Scale bars: 50 μm.

Given the importance of TNF levels in IBD as well as many other diseases (43, 44), we next aimed to determine the clinical relevance of our findings. In IBD, goblet cell numbers have been reported to decrease (23, 45), while anti-TNF treatment has been shown to lead to an increase in goblet cell numbers in mouse colitis (46). Here, we showed that loss of TNF causes an increase in goblet cell number under homeostatic conditions. Thus, we set out to determine the effect of TNF under conditions that mimicked homeostasis and disease in patients. We obtained noninflamed ileal tissue samples from IBD patients, IBD patients undergoing anti-TNF therapy, and healthy donors, and quantified the goblet cell proportion in crypt epithelium (Figure 6). We found that noninflamed tissue from IBD patients and healthy donors had a similar proportion of goblet cells, which supports the notion of noninflamed IBD tissue as a good model of normal goblet cell homeostasis in patients. Noninflamed IBD tissue thus allowed for the analysis of the effect of anti-TNF therapy in a condition resembling the steady state in humans.

Strikingly, IBD patients undergoing anti-TNF therapy had a higher proportion of crypt goblet cells than IBD patients and healthy donors (Figure 6, B and C). This reproduces the goblet cell hyperplasia phenotypes in TNF loss-of-function mice (Figures 2G and 4F), suggesting a mechanism of TNF control of goblet cell differentiation in human physiology similar to that in mouse.

## Discussion

Mucin provides a layer of protection against external pathogens and contaminants (5, 6). In the respiratory tract, the epithelium is protected from pathogens in part by active ciliary movement that clears mucin away from the epithelium. In contrast, the digestive tract lacks ciliary movement but still maintains flux of mucin away from the epithelium (5). In healthy intestines, the mucus layer facilitates waste expulsion, protects the host against pathogens, and houses commensal microbes that aid in nutrient absorption (2, 47). However, mucus can accumulate in disease states, leading to increased bacterial load, inflammation, and tissue damage. Loss of the mucosal barrier can also lead to disease by induction of persistent intestinal inflammation, as seen in IBD (48). Thus, a balance between the maintenance and turnover of mucus is essential for gut homeostasis. However, the upstream molecular mechanisms regulating the production and flux of mucin are poorly understood.

While TNF is a cytokine with a well-appreciated role in inflammation, here, we revealed previously underappreciated functions of TNF in epithelial mucin homeostasis. First, TNF regulates mucin production by regulating the differentiation of goblet cells, the mucin-producing cells in the respiratory and digestive tracts. Both TNFR1 and TNFR2 contribute to changes in goblet cell number (Supplemental Figure 2, G and H), and therefore, both may also have roles in regulating differentiation. Second, TNF controls the flux of mucin along the epithelial surface through TNFR1 by PKC-dependent induction of CFTR activity. These newly identified roles expand our understanding of the functional repertoire of TNF.

Developmental and in vitro studies have shown that TNFR2 signaling promotes transcription of *Muc2* (15, 19). Our work in adult mice found that TNF does not affect MUC2 protein expression but rather suppresses goblet cell differentiation, the primary cell type tasked with mucin biosynthesis. Therefore, changes in *Muc2* expression levels in other systems may reflect changes in the number of *Muc2*-expressing cells, as opposed to changes in transcript levels on a per-cell basis. While our studies show that loss of TNF function expands the number of ATOH1^+^ cells, another group has shown that TNF can stabilize ATOH1 in the context of colon cancer (49). This may suggest that TNF has different roles in the colon during cancer, when compared with the small intestine during homeostasis. Our expression analysis revealed that both TNF and TNFR2 are enriched in the adult crypt, where stem cells and progenitors are localized. We also observed increased numbers of secretory progenitors and goblet cells when TNF is lost. Taken together, these data indicate that TNF signaling functions as a regulator of goblet cell differentiation in the small intestine.

A number of studies have described TNF as a prodifferentiation factor of immune cells (50), skeletal muscle cells (51), and bone cells (52). Furthermore, NF-κB, which is a downstream effector of TNF signaling, is important for the Paneth versus goblet cell fate decision. Genetic ablation of NF-κB signaling in the intestine leads to a significant decrease in Paneth cell numbers, defective Paneth cell maturation, and an increase in goblet cell number (53). We identified TNF as a differentiation factor in the intestine that has no effect on Paneth cell number but does control goblet cell number. This suggests that TNF may be acting independently of NF-κB or on tangential signaling pathways in addition to NF-κB. Both Wnt and Notch signaling have been shown to be important for cell fate decisions in the intestine (26, 54) and TNF has been shown to promote Wnt and Notch signaling in certain contexts (55, 56). An intriguing hypothesis is that TNF acts together with Notch to regulate goblet cell differentiation in the intestine.

Our observation that TNF controls goblet cell number is supported by several reports. Mouse studies have shown that TNF promotes the loss of goblet cells (15, 18, 57), while clinical studies of IBD patient samples, who typically have elevated TNF levels, also revealed a lower number of goblet cells and partial loss of mucus barriers (22, 45). Consistent with these findings, anti-TNF treatment in mouse colitis causes goblet cell hyperplasia (46). Furthermore, in noninflamed human ileal samples we found that anti-TNF therapy results in an increased proportion of goblet cells. Like our mouse models, goblet cell hyperplasia in patients may promote increased accumulation of mucin, increased bacterial load, and prolonged gut transit. Our data highlight important considerations during anti-TNF therapy in IBD, mainly the collateral damage to surrounding noninflamed tissue, which may exacerbate inflammation.

Reduced goblet cell numbers in the context of elevated TNF have typically been interpreted as elevated goblet cell death. However, a recent RNA-seq analysis of IBD colon patient samples with elevated TNF (58) revealed that there is also a decrease in secretory transit-amplifying (TA) cells and immature goblet cells. Similarly, we found an increase in secretory progenitors at the expense of absorptive progenitors after the loss of TNF, accompanied by an increase in goblet cell number. Additionally, secretory lineage tracing and EdU pulse-chase experiments demonstrated no change in secretory cell turnover in the absence of TNF. These data may clarify previous reports by indicating that TNF controls goblet cell numbers by regulating cell lineage choice rather than cell death alone.

In organoids, we discovered that the pleiotropic effects of TNF on mucin homeostasis include induction of CFTR channel activity and flow, resulting in inflation of organoid lumens. TNF regulates the CFTR-dependent inflation-collapse dynamics observed in organoids, which is an important contributor to organoid cell patterning and morphogenesis (39, 59). A link between TNF and CFTR is also suggested by studies of human bronchial epithelial and HeLa cell monolayer cultures, in which TNF promotes CFTR apical localization and boosts cell chloride currents (42). Our studies are consistent with this body of work, suggesting a general mechanism of TNF modulation of CFTR activity through TNFR1 across various organs, including the intestine. Interestingly, pathologies associated with TNF or CFTR dysfunction exhibit several phenotypic similarities. For example, *Cftr*-knockout mice exhibit increased mucin, slower gut transit, and increased bacterial load (8, 24). In addition, patients with cystic fibrosis are 7 times more likely to have IBD, a disease characterized by elevated levels of TNF (60). This association implies that the phenotypes seen during cystic fibrosis may lead to IBD and thus provides clinical support for a model where increased goblet cells and mucin in patients undergoing anti-TNF therapy exacerbates inflammation and IBD.

In conclusion, the data presented here demonstrate that epithelial TNF is an essential regulator of mucin production and flux in the intestine. In addition to the canonical functions of TNF in inflammation and cell death, we reveal roles for TNF in intestinal cell differentiation and modulation of CFTR activity. Our findings provide new considerations for the homeostatic roles of TNF in healthy tissue in developing future interventions for IBD using anti-TNF therapy.

## Methods

### Mice.

*Tnf^−/−^* (stock 005540; ref. 23), *Rosa26^tdTomato^* (stock 007905; ref. 61), *Tnfr1^−/−^* (stock 003242; ref. 62)*, Tnfr1^−/−^ Tnfr2^−/−^* (stock 003243; ref. 62), and *Vil^CreERT2^* (stock 020282; ref. 34) mice were purchased from the Jackson Laboratory and maintained on a C57BL/6 background. *Tnfr2^−/−^* mice were generated by backcrossing *Tnfr1^−/−^ Tnfr2^−/−^* mice with C57BL/6 mice. Additional mouse lines include combinations of the following alleles or transgenes: *Lgr5^DTR-GFP^* (63), *Atoh1^CreERT2^* (25), *Cftr^fl^* (8), and *Tnf^fl^* (35). All mice were between the ages of 14 and 20 weeks at the time of experiments.

### Tamoxifen induction.

Tamoxifen (T5648-5g, Sigma-Aldrich) was dissolved in corn oil and administered via oral gavage at a dosing of 1 mg tamoxifen/kg of body weight for each mouse. For conditional knockout studies, mice were given a single dose of tamoxifen each day by oral gavage for 6 consecutive days. To label secretory progenitors, mice were given a single dose of tamoxifen at the start of the experiment, and tissue was harvested at either 36 hours or 72 hours after induction.

### Tissue preparation for mucin staining.

For mucin staining, the ileum (distal third of small intestine) was isolated. Fragments of 1.5 cm in length containing fecal pellets were then isolated and placed directly into methacarn fixative (60% methanol, 30% chloroform, 10% acetic acid). Fragments were fixed for 7 days with rocking at room temperature. Following fixation, fragments were washed twice in methanol for 20 minutes per wash, twice in absolute ethanol for 20 minutes each, and twice in xylene for 10 minutes each. The tissue was then embedded in paraffin blocks following standard procedures and cut into 4-μm sections.

### Tissue preparation for immunofluorescence and immunohistochemistry.

Animals were anesthetized via intraperitoneal (i.p.) injection of 250 mg/kg of body weight Avertin (2,2,2-tribromoethanol) and transcardially perfused with 4% paraformaldehyde (PFA) diluted in 1× PBS. Tissues were postfixed for 24 hours at 4°C and processed for paraffin embedding following standard procedures.

### Tissue preparation for RNAscope.

The ileum was isolated and immersion fixed in 4% PFA for 24 hours at room temperature. The tissue was then processed for paraffin embedding following standard procedures.

### RNAscope in situ hybridization.

RNA in situ hybridization was performed on 7-μm sections using RNAscope 2.5 High Definition (HD) – Red Assay (Advanced Cell Diagnostics, 322350) to assess *Tnf* (catalog 311081), *Tnfrsf1a* (*Tnfr1*, catalog 438941), and *Tnfrsf1b* (*Tnfr2*, catalog 438951) expression and using the RNAscope 2.5 HD Duplex Reagent Kit (Advanced Cell Diagnostics, 322430) to assess *Dll1* (catalog 425071) and *Cftr* (catalog 483011) expression. The assay was performed based on the manufacturer’s protocol, with 15 minutes of target retrieval and 30 minutes of protease digestion.

### Immunofluorescence and immunohistochemistry.

Slides containing 7-μm sections for mouse and 4-μm sections for human from paraffin-embedded tissue were heated at 60°C for 30 minutes until the wax was melted. After rehydration, antigen retrieval was performed using sodium citrate buffer pH 6.0 or Tris-EDTA pH 9.0 in a pressure cooker for 15 minutes. Sections were then blocked for 2 hours at room temperature in 5% normal goat serum (005-000-121, Jackson ImmunoResearch). Next, slides were incubated in primary antibody overnight at 4°C at the indicated concentrations. The next day, tissues were stained in secondary antibody for 2 hours at room temperature at the indicated concentrations. Finally, slides were stained with DAPI (5 μg/mL; D9542, Sigma-Aldrich) for 20 minutes at room temperature and mounted in Prolong Gold Antifade Medium (P36930, Thermo Fisher Scientific). Primary antibodies were used at the following dilutions: rat anti–E-cadherin (1:200; 13-1900, Thermo Fisher Scientific), rabbit anti–E-cadherin (1:350; 3195s, Cell Signaling Technology), mouse anti–E-cadherin (1:250, 610181, BD Biosciences), rabbit anti-lysozyme (1:500; A0099, Dako), rabbit anti-MUC2 (1:250; NBP1-31231, Novus Biologicals), rabbit anti–TNF-α (1:250; ab6671, Abcam), rat anti–TNF-α (1:250, 506302, BioLegend), rabbit anti-TNFR1 (1:250; PA5120358, Thermo Fisher Scientific), rabbit anti-TNFR2 (1:250; PA5-80159, Thermo Fisher Scientific), rabbit anti-RFP (1:250; 600-401-379, Rockland), and chicken anti-RFP (1:250; 600-901-379, Rockland).

Secondary stains and antibodies were used at the following dilutions: FITC-conjugated *Ulex* *europaeus* agglutinin I (UEA-1–FITC) (50 μg/mL; L9006-1 mg, Sigma-Aldrich), UEA-1–rhodamine (1:250; RL-1062-2, Vector Laboratories), Alexa Fluor–conjugated secondary antibodies (1:250; A-11034, A-21429, A-21245, A-21247, A-21432, Thermo Fisher Scientific), and goat biotinylated secondaries (1:250–1:1000; BA-1000-1.5, BA-9200-1.5, Vector Laboratories)

For immunostaining requiring signal amplification, TSA Cy3 Amplification kits were used (SAT704A001EA, PerkinElmer) and manufacturer-provided protocols were followed.

### Intestinal transit assay.

Mice were fasted 24 hours prior to tissue harvest and given free access to water. Sterile saline solution (0.9% NaCl) was used to resuspend 70 kDa FITC-dextran (46945, Sigma-Aldrich) at a concentration of 25 mg/mL. Each mouse was administered 0.5 mg (20 μL) of 70 kDa FITC-dextran solution by oral gavage for 1 hour. The gastrointestinal tract from the stomach to the rectum was then harvested and imaged on a Bio-Rad ChemiDoc Touch Imaging System using a SYBR Green filter. Displacement of FITC was measured starting at the stomach-duodenum junction.

### Bacterial load 16S rRNA qPCR.

Fecal pellets were harvested from the ileum of mice and immediately stored at –20°C. gDNA was later isolated using the Qiagen DNeasy PowerLyzer PowerSoil Kit following the manufacturer’s protocol. qPCR targeting the universal bacterial 16S rRNA gene was then performed on isolated gDNA using universal 16S primers (64) and primers targeting mouse genomic *Gapdh*. Relative 16S copies were calculated using the ΔΔCt method by normalizing 16S Ct values to mouse genomic *Gapdh* Ct values, similar to what has been reported previously (65).

### Small molecules and recombinant proteins.

Mouse recombinant TNF-α (410-MT-025/CF, R&D Systems) was resuspended in PBS with 0.1% BSA at a concentration of 100 μg/mL and stored at –80°C. CFTRinh-172 (C2992, MilliporeSigma) was resuspended in DMSO at a concentration of 50 mM and stored at –80°C. Forskolin (6886-10MG, Sigma-Aldrich) was resuspended in DMSO at a concentration of 50 mM and stored at –80°C. (*Z*)4-Hydroxytamoxifen (74056, Stemcell Technologies) was resuspended in DMSO at a concentration of 25 mM and stored at –80°C. Bisindolylmaleimide I (GF109203X; S7208, Selleckchem) was resuspended in DMSO at a concentration of 10 mM and stored at –80°C. EGF (53003-018, Invitrogen) was resuspended in PBS with 0.1% BSA at a concentration of 50 μg/mL and stored at –20°C. Noggin (6057-NG-100/CF, R&D Systems) was resuspended in PBS with 0.1% BSA at a concentration of 100 μg/mL and stored at –20°C. Y-27632 (125410, Tocris) was resuspended in DMSO at a concentration of 10 mM and stored at –20°C. Jagged Chimera (AS-61298, Anaspec) was resuspended in sterile MilliQ H_2_O at a concentration of 1 mM and stored at –20°C.

### Organoid tissue culture.

Mouse intestinal organoid cultures were established from ileal segments isolated from mice aged 12–20 weeks, according to established protocols (66). Briefly, following 15 minutes of incubation in intestine harvest buffer, filleted intestinal segments were placed in crypt dissociation buffer and rocked on ice for 1 hour. Crypts were filtered through a 70-μm mesh filter and plated in a preheated 24-well plate in 50 μL Matrigel (356231, Corning) domes supplemented with 10% R-Spondin–conditioned medium, Jagged Chimera (10 μM), Noggin (100 ng/mL), and EGF (50 ng/mL). After Matrigel domes solidified, organoids were overlaid with 500 μL ENR (EGF/Noggin/R-Spondin) medium supplemented with Y-27632 (10 μM; 125410, Tocris). After 48 hours, medium was changed to ENR medium alone. From this point forward, organoids were cultured in standard ENR.

### 4-Hydroxytamoxifen induction of Cftr–conditional knockout organoids.

5 days after passaging *Villin^CreERT2^*; *Cftr^fl/fl^* organoids were treated with 1 μm 4-hydroxytamoxifen for 48 hours to induce knockout, and then passaged and plated for experiments. For swelling experiments, induced organoids were treated with 1 ng/mL rTNF immediately after plating and the relative lumen area was measured at 24 hours. For qPCR validation of *Cftr* knockout, induced organoids were grown in normal media for 4 days after plating and RNA was harvested for qPCR.

### Live imaging of organoids.

Ileal organoids were passaged on day 7 in culture by mechanical dissociation and plated in a 96-well plate format. Organoids were plated at a density of 50 organoids per 3 μL Matrigel droplet. After the droplet solidified, each well was overlaid with 200 μL ENR medium supplemented with the appropriate drug treatments. The plate was immediately transferred to a Zeiss Cell Observer confocal microscope with a Yokogawa CSU-X1M spinning disc system and incubation chamber. The system was pre-equilibrated for 30 minutes to 37°C and 5% CO_2_. Images were acquired every 20 minutes for 48 hours, at ×5 magnification using brightfield transluminescence. A 90-μm *Z*-stack was acquired for each region (*Z*-step = 30 μm).

### Forskolin-induced swelling assay.

Organoids were passaged into a 96-well plate at a density of 50 organoids per well and allowed to grow in normal ENR media for 2 days. At the start of the experiment, old media were replaced with 100 μL of fresh media containing 1 ng/mL rTNF, 20 μM CFTRinh-172, and 5 μM GF109203X. The plate was then placed back in the incubator for 4 hours at 37°C and 5% CO_2_. Afterward, 100 μL of media containing additives plus 0.8 μM forskolin was quickly added to wells to yield a total well volume of 200 μL and a final concentration of 0.4 μM forskolin. The plate was immediately transferred to a Zeiss Cell Observer spinning disc confocal system with Yokogawa CSU-X1M and incubation chamber. The system was pre-equilibrated for 30 minutes to 37°C and 5% CO_2_. Tiled images of the center plane of each well were acquired every 10 minutes for 120 minutes at ×5 magnification.

### Imaging of tissue sections.

Image acquisition of immunostained intestinal swiss rolls used for quantitative analysis was performed on a Leica DMi8 inverted microscope at ×20 magnification. Image acquisition of representative regions of immunostained slides was performed using a Zeiss LSM 900 and a ×40 objective. A 4- or 7-μm *Z*-stack was acquired for each region (*Z*-step = 1 μm).

### Human intestinal tissue.

Healthy human adult intestinal tissue was obtained via an IRB-approved research protocol with the organ procurement organization for Northern California, Donor Network West, in collaboration with UCSF Viable Tissue Acquisition Lab (VITAL) Core. At the time of organ acquisition for clinical transplantation, intestinal tissue was also collected from an organ donor who consented to research. After the clinical procurement process, full-length intestinal tissue was stored and transported in University of Wisconsin preservation media on ice. As tissues are from deidentified, deceased individuals lacking associated health information, the study is IRB designated as non–human subjects research. Specimens from IBD and nonaffected individuals were obtained from standard-of-care archives, facilitated through Cedars-Sinai Medical Center MIRIAD IRB 3358.

### Statistics.

Normally distributed data were analyzed using parametric 2-tailed Student’s *t* test with Welch’s correction unless otherwise noted. The nonparametric Mann-Whitney *U* test was used if the data did not fit a normal distribution. A *P* value of less than 0.05 and a confidence interval of 95% were considered significant. Data are presented as mean ± SD for parametric data or as median ± interquartile range for nonparametric data. For organoid rTNF and CFTRinh-172 treatment studies, 1-way ANOVA with Dunnett’s multiple-comparison test was used to compare all treatment groups to the control group. Statistical information is otherwise provided in the figure legends.

### Study approval.

Mice were maintained in the animal facility at UCSF in compliance with all ethical guidelines established by the Institutional Animal Care and Use Committee (IAUCUC) and Laboratory Animal Resource Center (LARC). All experimental procedures were approved by the Laboratory Animal Resource Center at UCSF.

### Data availability.

Values for all data points are available in the Supporting Data Values file.

## Author contributions

EAR, DCA, JR, ZJG, and ODK conceived studies and designed experiments. EAR, DCA, and JR conducted experiments. EAR, DCA, and JR analyzed and interpreted the data. TW performed FACS experiments and analysis. RKZ and BP processed and shared human donor ileal histological samples. AM and SR generated the histological panel of IBD patient samples. ARG and JMG procured human adult intestinal tissue from donors. DB provided bioinformatics support. EAR, DCA, and JR wrote the manuscript. All authors contributed to editing and review of the manuscript.

## Supplementary Material

Supplemental data

Supporting data values

## Version 1 08/29/2023

In-Press Preview

## Version 2 10/16/2023

Electronic publication
