# Supplementary material for: Epithelial TNF controls cell differentiation and CFTR activity to maintain intestinal mucin homeostasis
Source: J Clin Invest. 2023 Oct 16;133(20):e163591. doi: 10.1172/JCI163591 (PMC10575728; doi:10.1172/JCI163591)
Supplement: Supplemental data [file jci-133-163591-s007.pdf]

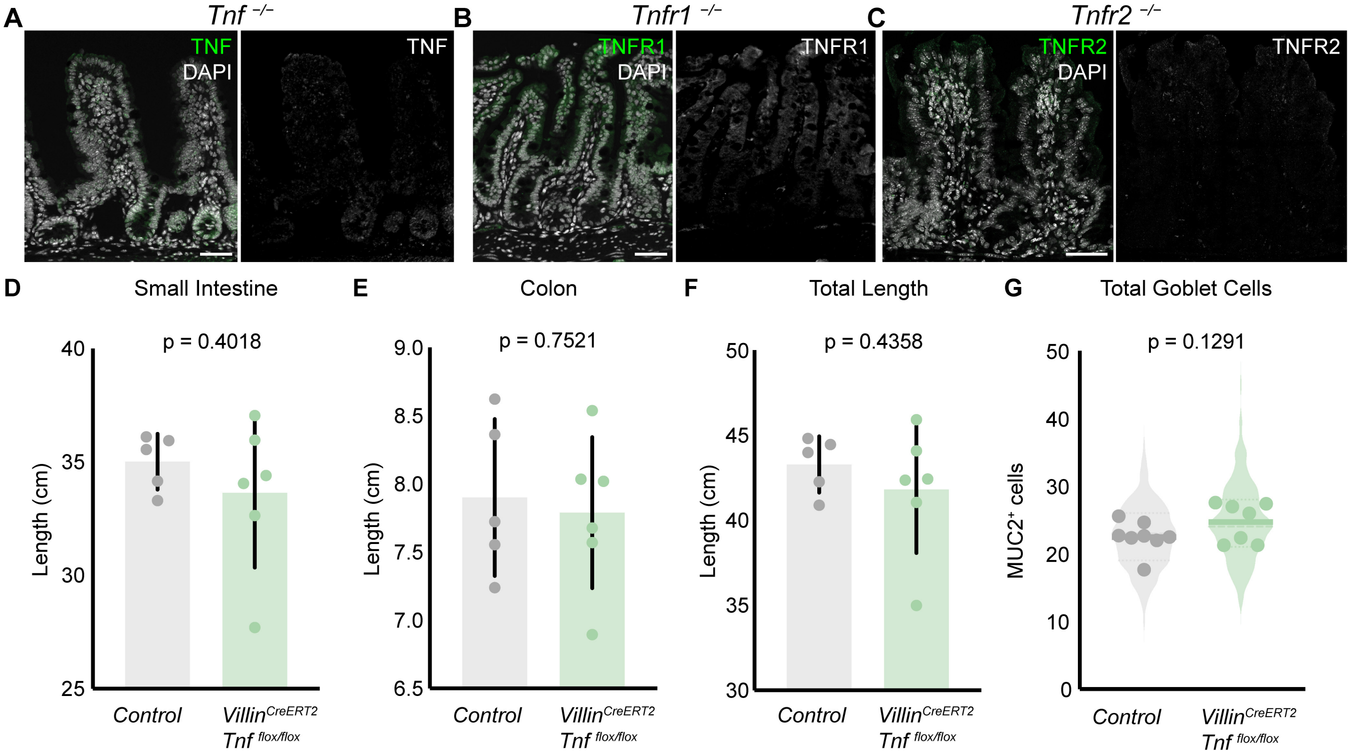

Supplemental Figure 1. (A-C) Validation of TNF, TNFR1, and TNFR2 immunostaining specificity. Protein expression in control mice was compared to relevant knockout mice. (A) *Tnf* $^{-/-}$ mice lose expression of TNF. (B) *Tnfr1*$^{-/-}$ mice lose expression of TNFR1. (C) *Tnfr2*$^{-/-}$ mice lose expression of TNFR2. (D-F) Full-length intestine (stomach to rectum) from control or *Villin*^{CreERT2}*; Tnf*$^{flox/flox}$ mice was isolated, photographed, and digitally measured. N = 5 control, 6 *Villin*^{CreERT2}*; Tnf*$^{flox/flox}$ mice. (D) Small intestine length was defined as the segment starting from the end of the stomach to the beginning of the cecum. (E) Colon length was defined as the segment starting after the cecum and ending at the rectum. (F) Total length was defined as the entire isolated segment (stomach to rectum). (G) Quantification of total MUC2^{+} goblet cells. N = 8 control , 7 *Villin*^{CreERT2}*; Tnf*$^{flox/flox}$ mice. At least 25 crypt-villus units per mouse were counted. p-value calculated by unpaired two-tailed t-test for panels D-G.

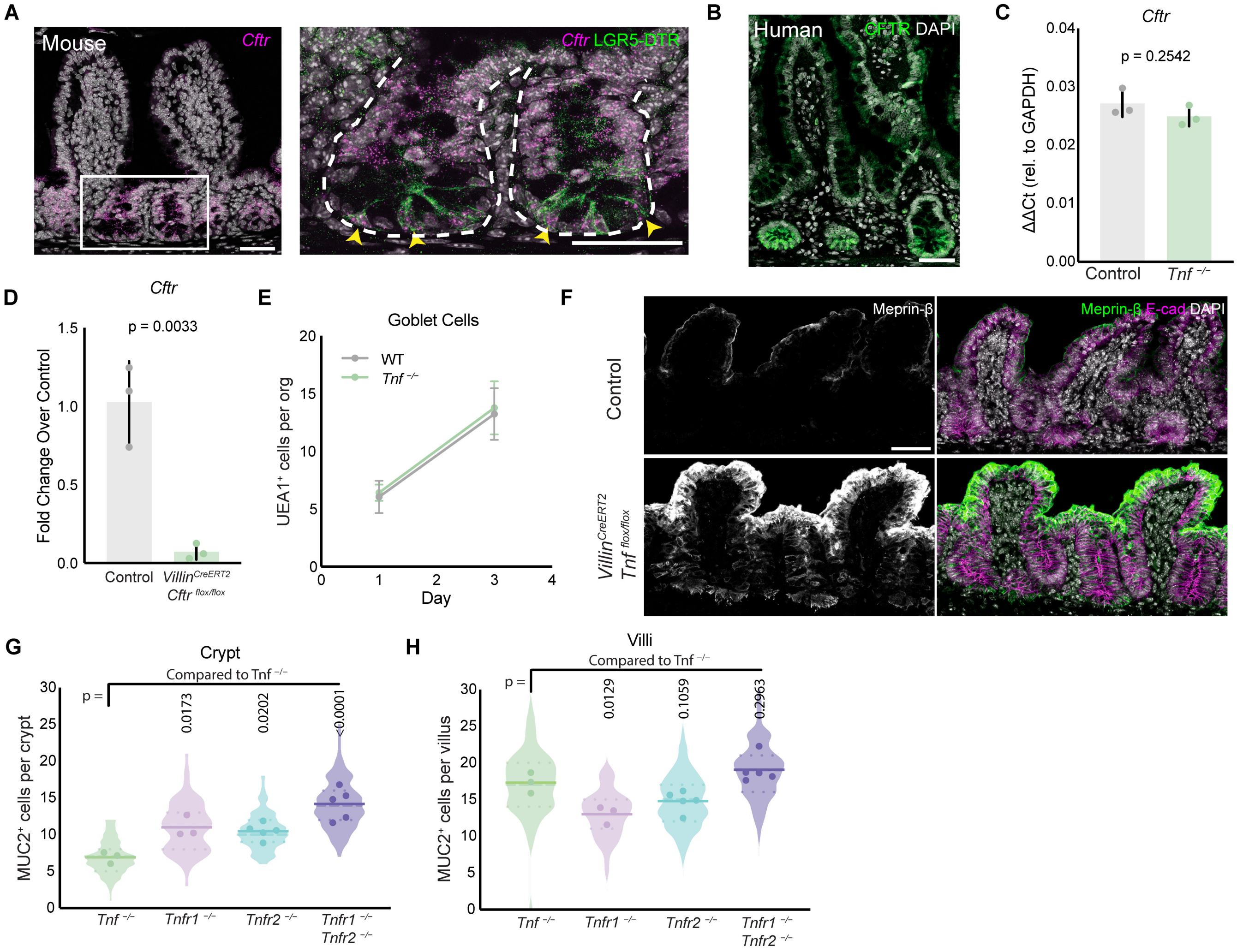

Supplemental Figure 2. (A) RNAscope of *Cftr* transcripts and immunofluorescence staining of LGR5-DTR in mouse intestine. Scale Bar= 50 μm. White box outlines inset. Dotted line marks epithelial-mesenchymal border. N = 3 LGR5-DTR mice. Yellow arrows mark LGR5+ stem cells with *Cftr* expression. (B) Expression of CFTR in healthy human donor showing enrichment in epithelial crypts. Scale Bar= 50 μm. (C) qPCR analysis of *Cftr* expression in organoids 24 h after passaging. N = 3 control, 3 *Tnf* $^{-/-}$ organoid lines. (D) 4-OHT treatment results in significant loss of *Cftr* in *Villin*^{CreERT2}*; Cftr*$^{flox/flox}$ *(Cftr cKO)* organoids. N = 3 control, 3 *Cftr cKO* organoid lines. (E) WT and *Tnf* $^{-/-}$ organoids have comparable numbers of goblet cells at 1 day and 3 days post-passaging. N = 3 WT, 3 *Tnf* $^{-/-}$ organoid lines. (F) Immunofluorescence of meprin-β in the intestine. N = 6 control, 7 *Tnf cKO* mice. Scale Bar= 50 μm. (G) TNFR1 and TNFR2 both contribute to goblet cell hyperplasia in intestinal crypts. (H) TNFR1 loss leads to reduced goblet cell number in intestinal villi. N = 3 *Tnf* $^{-/-}$, 3 *Tnfr1*$^{-/-}$, 5 *Tnfr2*$^{-/-}$, 5 *Tnfr1*$^{-/-}$ *Tnfr2*$^{-/-}$ mice. At least 25 crypt-villus units per mouse were quantified. p-value calculated by unpaired two-tailed t-test for panels C-D. A p-value was calculated by ordinary one-way ANOVA analysis with Dunnett's multiple comparisons test comparing all groups to the *Tnf* $^{-/-}$ group for panels G and H.
